# Longitudinal cardiac remodeling in collegiate American football players as assessed by echocardiography during their collegiate career

**DOI:** 10.1002/clc.24121

**Published:** 2023-08-13

**Authors:** Robert F. Hamburger, Yasmeen Taha, Mohammed Ruzieh, James R. Clugston, Eileen M. Handberg, Fred Reifsteck, Matthew W. Martinez, Carl J. Pepine, Katherine M. Edenfield

**Affiliations:** ^1^ Division of Cardiovascular Medicine University of Florida College of Medicine Gainesville Florida USA; ^2^ Division of Cardiology Malcom Randall VA Medical Center Gainesville Florida USA; ^3^ Department of Medicine University of Florida College of Medicine Gainesville Florida USA; ^4^ Department of Community Health and Family Medicine University of Florida College of Medicine Gainesville Florida USA; ^5^ Department of Sports Medicine University of Georgia Athens Georgia USA; ^6^ Division of Cardiology Atlantic Health System Morristown New Jersey USA

**Keywords:** athlete, cardiac remodeling, echo, football

## Abstract

**Background:**

Studies on the longitudinal effects of intense physical training on cardiac remodeling are limited, especially in American collegiate football players.

**Hypothesis:**

College‐level American football training will result in remodeling in a pattern consistent of a sport with moderate static and dynamic demands with increases in both wall and chamber sizes.

**Methods:**

We studied 85 American collegiate football players who underwent transthoracic echocardiogram (TTE) for asymptomatic or mild COVID‐19‐related illness and compared the changes in echo dimensions to their preparticipation screening TTE. Pre‐ and posttraining variables were compared using a paired *t*‐test for normally distributed variables.

**Results:**

Mean age was 19 years ± 1 and 61% of athletes were Black. Mean follow‐up between TTEs was 21 ± 13 months. There was an increase in left atrial volume index (26.4 ± 5.5 to 32.8 ± 8.4 mL/m^2^, *p* < .001), LV end diastolic diameter (5.13 ± 0.4 to 5.27 ± 0.4 cm, *p* = .003), basal RV diameter (3.28 ± 0.7 to 3.83 ± 0.5 cm, *p* = <.001), LV mass index (86.7 ± 15.3 to 90.1 ± 15.3, *p* = .015), and aortic root diameter (3.1 ± 0.4 to 3.2 ± 0.3 cm, *p* = .03) from pre‐ to posttraining, with a slightly greater magnitude in athletes with >2 years of training. Presence of left atrial enlargement (≥35 mL/m^2^) increased from 2.9% to 29% pre‐ to postparticipation in athletes with >2 years training. No significant changes in wall thickness, diastolic function, or right ventricular systolic function were observed.

**Conclusion:**

American football players college‐level training was associated with increases in left and right ventricular chamber sizes, left atrial size, and aortic root diameter.

## Graphical abstract

Participation in an American collegiate football training program resulted in increases in left atrial volume index (LAVI), LV Mass index, LV end diastolic and systolic diameters (LVEDD, LVESD), basal RV diameter (RVID), and aortic root diameter (ARD) over a mean follow‐up of 21 ± 13 months.

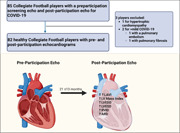

## 1 BACKGROUND

Preparticipation cardiovascular screening of athletes is common practice globally. In addition to physical exam and resting electrocardiograms (ECGs), echocardiography is used in many centers as a first line imaging modality to screen for evidence suggesting cardiovascular disease.^1^ The term “athlete's heart” has been used to describe the morphological changes seen in trained athletes.^2^ This cardiac remodeling generally varies by the training type (endurance, strength, or mixed) and the duration of training.^3^ These changes are often described in elite athletes; however, few recent studies have shown that similar changes can be observed in collegiate and adolescent athletes.^4^, ^5^ The adaptive cardiac response differs by the training type. In endurance athletes, an increase in the size of cardiac chambers and variable degrees of eccentric left ventricular (LV) hypertrophy are seen compared to strength‐training athletes who develop increased LV mass and concentric hypertrophy.^3^

American football involves different field positions, and the training is usually a mixture of both endurance and strength exercise. The longitudinal effects of intense training on cardiac remodeling are not well described in American football especially in collegiate athletes. This study aims to describe the longitudinal cardiac remodeling seen in association with multiple years of participation in collegiate American football by comparing routine preparticipation transthoracic echocardiograms (TTEs) performed during initial assessments to TTEs performed later in their collegiate careers.

## 2 METHODS

We studied all collegiate football players from the University of Florida and the University of Georgia who had both a TTE at the time of their matriculation to their respective university and a subsequent TTE at a later point in their collegiate career at the institution (total *N* = 85). The reason for the second TTE was that these athletes were infected with SARS‐CoV‐2 (COVID‐19) between June 2020 and August 2021 and underwent a TTE after their infection, which was mandated as part of the COVID‐19 return to play protocol for both Universities at the time. One athlete with a diagnosis of hypertrophic cardiomyopathy discovered on pre‐participation physical examination (PPE) and echocardiogram was excluded. To minimize potential confounding influence of COVID‐19 on parameters of right heart function for the post‐COVID‐19 TTE, only athletes who had experienced mild (not requiring hospitalization, emergency room visit or special interventions) or asymptomatic disease were included. As such, two athletes were excluded—one with a pulmonary embolism and the other with pulmonary fibrosis on chest imaging. This left 82 otherwise healthy athletes with TTEs performed at two time points during their collegiate football career (henceforth referred to as pre‐ and postparticipation) and the comparison of these TTEs allowed for description of cardiac remodeling. All athletes had standard 12‐lead ECGs at their PPE as well as upon diagnosis of COVID‐19. The study was approved by the University of Florida Institutional Review Board and is in accordance with the Declaration of Helsinki.

TTE's were performed *en masse*by Athletic Heart^6^ during the routine PPEs using a Philips CX50 machine with an S5‐1 transducer with a 1–5 MHz frequency range (Philips Medical). Athletes who missed initial screening days had TTEs performed on a GE Vivid E9 echocardiography machine with an M5 cardiac probe at the institution's designated cardiology office. Repeat echocardiography after COVID‐19 was also performed similarly after resolution of COVID symptoms and at a minimum of 10 days since start of symptoms. All cardiac measurements were made according the American Society of Echocardiography chamber quantification guidelines.^7^ Left ventricular end systolic and diastolic diameter (LVESD and LVEDD), interventricular septal and posterior wall thickness (IVS and PW), and aortic root diameter (ARD) were measured in parasternal long axis views. Values were indexed to body surface area (BSA) where relevant. Relative wall thickness (RWT) was calculated as 2 × PWT/LVEDD and LV mass index as LV mass calculated by Devereux's formula ([0.8 × 1.04 [(LVEDD + interventricular septum + posterior wall thickness)^3^ − (LVEDD)^3^]] + 0.6 g) indexed to the BSA. Left atrial volume index (LAVI) and left ventricular ejection fraction (LVEF) were performed by a biplane method of disks in the apical four‐ and two‐chamber views. Right ventricular internal diameter (RVID) was performed in an apical four‐chamber RV‐focused view at the basal RV. Tricuspid annular plane systolic excursion (TAPSE) was measured using M‐mode echocardiography of the tricuspid annulus. Diastolic parameters including mitral valve E (MV E), A, and E/A were performed using pulsed wave Doppler at the tips of the mitral valve and e’ values were obtained using tissue Doppler of the lateral and medial mitral annulus. All TTEs were analyzed for clinical purposes by cardiologists at the institutions who routinely read athlete TTEs.

The training regimen of these collegiate football players is fairly heterogeneous and is a combination of aerobic training such as long‐distance running and anerobic training including sprinting, field work that would include blocking and tackling drills, and weight training. This is performed in an on‐field practice setting and an off‐field strength and conditioning program. The off‐field strength and conditioning program is 6 h per week for 8 weeks (40 h/year) in the off‐season and 3 h per week for 13 weeks (39 h/year) in‐season. The on‐field practice schedule includes five 2‐h practices per week (130 h/year) in‐season, a 3‐week postseason practice period of approximately 30 h total, and a fall camp of 4 weeks with about 40 h total. Time spent on anerobic versus aerobic training differs slightly for line position (offensive and defensive line) compared to non‐line position athletes. Lineman perform approximately 10%–20% aerobic and 80%–90% anaerobic training whereas non‐line athletes perform approximately 20%–30% aerobic and 70%–80% anaerobic training. Statistical analysis was performed using the SPSS V.28 statistical package (IBM). All data were summarized using descriptive statistics. Means and standard deviations were used to describe normally distributed variables while median and interquartile ranges were reported for all non‐normally distributed variables. Pre‐ and posttraining variables were compared using a paired *t*‐test for normally distributed variables and using the Wilcoxon signed rank test for non‐normal variables.

## 3 RESULTS

Pertinent demographic data are summarized in Table 1. Mean age was 19 years ± 1 and the majority of athletes were Black (61%). Mean BSA was 2.31 ± 0.3 m^2^. The majority of athletes played non‐line positions (*n* = 51, 62.2%) compared to line positions (i.e., offensive or defensive line).

**Table 1:** Baseline demographic information of the study population.

	Mean ± SD*
Age, year	19 ± 1
Heart rate, bpm	62 ± 11
Height, cm	188 ± 7
Weight, kg	103 ± 25
BMI	29.06 ± 5.67
BSA, m^2^	2.31 ± 0.3
Years of training	1.8 ± 1.2
Systolic blood pressure, mmHg	128 ± 11
Diastolic blood pressure, mmHg	75 ± 7
Football position, *n* (%)	
Line position	31 (37.8)
Non‐line position	51 (62.2)
Race, *n* (%)	
Black	50 (61)
White	30 (36.6)
Asian	1 (1.2)
American Indian/Alaska Native	1 (1.2)

*Note*: Demographics represent mean ± *SD* except for specified categorical data represented as counts (*n*) and proportions (%).

Abbreviations: BMI, body mass index; BSA, body surface area.

**Figure 1:**
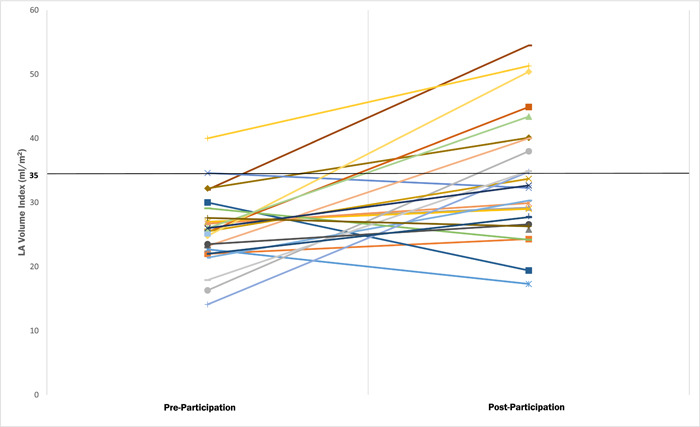
LAVI pre‐ and postparticipation in athletes with at least 2 years of participation. Horizontal line represents the cutoff for left atrial dilation. LAVI, left atrial volume index.

TTE parameters for all athletes pre‐ and posttraining are shown in Table 2. Mean follow‐up time between studies was 21 ± 13 months. There was a statistically significant increase in LAVI (26.4 ± 5.5 to 32.8 ± 8.4, *p* < .001), LVEDD (5.13 ± 0.4 to 5.27 ± 0.4, *p* = .003), LVESD (3.35 ± 0.3 to 3.47 ± 0.3, *p* = .004), RVID (3.28 ± 0.65 to 3.83 ± 0.48, *p* = <.001), LV Mass index (86.7 ± 15.3 to 90.1 ± 15.3, *p* = .015), and ARD (3.1 ± 0.35 to 3.2 ± 0.31, *p* = .03) from pre‐ to postparticipation. Table 3 shows TTE parameters for athletes with at least 2 years of training between studies (1‐year data is available in a supplemental table online). Results were similar but with a slightly greater magnitude of increase in LAVI, LVEDD, LVESD, LV mass index, RVID, and ARD from pre‐ to postparticipation. There was also a small but statistically significant decrease in LVEF (60.7 ± 3.6 to 58.9 ± 2.8, *p* = .007). There were no changes in parameters of wall thickness or diastolic function from pre‐ to postparticipation, regardless of duration between TTE. Of athletes with 2 or more years of training, there was only one (2.9%) with preparticipation left atrial enlargement, defined as a LAVI ≥ 35 mL/m^2^, which increased to 10 (29.4%) postparticipation (Figure 1).^7^ Table 4 shows comparison of TTE parameters for athletes who played line positions. Both positions showed increases in LAVI and RVID. Athletes playing line positions had an increase in LVEDD. Those playing non‐line positions had small increases in LVESD and ARD as well as LV mass index. However, there was no statistically significant change in any TTE parameter when specifically accounting for position. There were no athletes with left atrial abnormality on their 12‐lead ECG at both pre‐ and postparticipation.

**Table 2:** Echo parameters pre‐ and postparticipation (*n* = 82).

	Preparticipation	Postparticipation	*p* value
LVEF (%)	60.3 ± 3.4	59.6 ± 2.7	.104^a^
LAVI (mL/m^2^)	26.4 ± 5.5	32.8 ± 8.4	**<.001**
LVEDD (cm)	5.13 ± 0.4	5.27 ± 0.4	**.003**
LVEDI (cm/m^2^)	2.23 ± 0.2	2.27 ± 0.2	.114
LVESD (cm)	3.35 ± 0.3	3.47 ± 0.3	**.004**
PW (cm)	1.04 ± 0.1	1.06 ± 0.1	.171
IVS (cm)	1.03 ± 0.1	1.01 ± 0.1	.333
RWT	0.41 ± 0.06	0.41 ± 0.05	.536
LV mass index (g/m^2^)	86.7 ± 15.3	90.1 ± 15.3	**.015**
RVID (cm)	3.28 ± 0.65	3.83 ± 0.48	**<.001**
TAPSE (mm)	24 ± 3	25 ± 3	.226
ARD (cm)	3.1 ± 0.35	3.2 ± 0.31	**.030**
Aortic root index (cm/m^2^)	1.35 ± 0.15	1.37 ± 0.15	.291
MV E	0.86 ± 0.16	0.85 ± 0.16	.518
MV A	0.46 ± 0.12	0.44 ± 0.11	.150
MV E/A	2.01 ± 0.65	2.07 ± 0.63	.456
E'Lat	17.6 ± 3.4	17.5 ± 2.9	.721
E'Med	12.0 ± 2.1	12.4 ± 2.9	.146
E/e’ Med	7.0 ± 1.4	6.9 ± 1.4	.480
E/e’ Lat	4.9 ± 1.1	4.9 ± 1.4	.503

*Note*: Bolded values are those that reached statistical significance (*p* = .05).

Abbreviations: ARD, aortic root diameter; IVS, interventricular septal thickness; Lat, lateral; LAVI, left atrial volume index; LVEDD, left ventricular end diastolic diameter; LVEF, left ventricular ejection fraction; LVESD, left ventricular end systolic diameter; Med, medial; MV, mitral valve; PW, posterior wall thickness; RVID, right ventricular internal diameter; RWT, relative wall thickness; TAPSE, tricuspid annular plane systolic excursion.

a Performed using Wilcoxon‐Ranks test as LVEF was non‐normally distributed.

**Table 3:** Echo parameters pre‐ and postparticipation for at least 2 years (*n* = 34).

	Preparticipation	Postparticipation	*p* value
LVEF (%)	60.7 ± 3.6	58.9 ± 2.8	**.007** a
LAVI (mL/m^2^)	25.1 ± 5.0	33.2 ± 9.3	**.001**
LVEDD (cm)	5.09 ± 0.46	5.31 ± 0.48	**.006**
LVEDI (cm/m^2^)	2.26 ± 0.25	2.32 ± 0.22	.104
LVESD (cm)	3.33 ± 0.33	3.51 ± 0.39	**.006**
PW (cm)	1.05 ± 0.14	1.07 ± 0.12	.272
IVS (cm)	1.03 ± 0.15	1.03 ± 0.15	.884
RWT	0.42 ± 0.07	0.41 ± 0.05	.444
LV mass index (g/m^2^)	87.9 ± 18.7	95.9 ± 17.6	**.025**
RVID (cm)	3.04 ± 0.53	3.84 ± 0.55	**<.001**
ARD (cm)	3.08 ± 0.37	3.22 ± 0.36	**.037**
Aortic root index (cm/m^2^)	1.37 ± 0.16	1.40 ± 0.14	.188
MV E (cm/s)	0.85 ± 0.17	0.83 ± 0.16	.581
MV A (cm/s)	0.45 ± 0.13	0.42 ± 0.11	.2
MV E/A	2.02 ± 0.73	2.11 ± 0.76	.602
E'Lat (cm/s)	17.4 ± 3.2	17.4 ± 3.3	.992
E'Med (cm/s)	11.9 ± 1.9	12.1 ± 2.1	.776
E/e’ Med	7.2 ± 1.1	7.2 ± 1.2	.842
E/e’ Lat	4.9 ± 1.0	5.0 ± 1.0	.982

*Note*: Bolded values are those that reached statistical significance (*p* = .05).

Abbreviations: ARD, aortic root diameter; IVS, interventricular septal thickness; Lat, lateral; LAVI, left atrial volume index; LVEDD, left ventricular end diastolic diameter; LVEF, left ventricular ejection fraction; LVESD, left ventricular end systolic diameter; Med, medial; MV, mitral valve; PW, posterior wall thickness; RVID, right ventricular internal diameter; RWT, relative wall thickness; TAPSE, tricuspid annular plane systolic excursion.

a Performed using Wilcoxon‐Ranks test as LVEF was non‐normally distributed.

**Table 4:** Echo parameters by line versus non‐line position.

	Line position (*n* = 31)			Non‐line position (*n* = 51)			*p* value for line vs. non‐line^a^
	Preparticipation	Postparticipation	*p* value	Preparticipation	Postparticipation	*p* value	
LVEF (%)	60.1 ± 3.3	59.7 ± 2.7	.55^b^	60.5 ± 3.5	59.6 ± 2.9	.13^b^	.62
LAVI (mL/m^2^)	25.7 ± 6.4	33.1 ± 7.5	**<.001**	26.7 ± 4.7	32.6 ± 8.9	**<.001**	.55
LVEDD (cm)	5.32 ± 0.4	5.52 ± 0.4	**.02**	5.01 ± 0.43	5.11 ± 0.36	.06	.39
LVESD (cm)	3.44 ± 0.3	3.58 ± 0.4	.06	3.3 ± 0.35	3.4 ± 0.32	**.03**	.74
LVEDI (cm/m^2^)	2.03 ± 0.2	2.10 ± 0.2	.052	2.36 ± 0.2	2.37 ± 0.2	.278	.25
PW (cm)	1.10 ± 0.1	1.11 ± 0.1	.5	1.01 ± 0.12	1.03 ± 0.11	.21	.26
IVS (cm)	1.10 ± 0.1	1.07 ± 0.1	.37	0.98 ± 0.12	0.98 ± 0.11	.64	.60
RWT	0.41 ± 0.05	0.41 ± 0.05	.42	0.41 ± 0.06	0.41 ± 0.05	.87	.61
LV mass index (g/m^2^)	87.72 ± 15.52	92.59 ± 15.86	.187	86 ± 15.28	89.89 ± 14.94	**.03**	.38
RVID (cm)	3.44 ± 0.63	4.03 ± 0.55	**.003**	3.19 ± 0.65	3.73 ± 0.41	**<.001**	.85
TAPSE (mm)	25 ± 3	25 ± 4	.57	23 ± 3	24 ± 2	.24	.69
ARD (cm)	3.3 ± 0.3	3.4 ± 0.3	.54	3.0 ± 0.3	3.1 ± 0.3	**.03**	.36
Aortic root index (cm/m^2^)	1.28 ± 0.1	1.28 ± 0.1	.49	1.40 ± 0.14	1.42 ± 0.13	.11	.44
MV E	0.86 ± 0.15	0.87 ± 0.14	.81	0.86 ± 0.17	0.84 ± 0.14	.28	.41
MV A	0.48 ± 0.12	0.44 ± 0.12	.24	0.45 ± 0.12	0.43 ± 0.11	.38	.63
MV E/A	1.91 ± 0.6	2.1 ± 0.61	.15	2.08 ± 0.66	2.08 ± 0.68	.99	.33
E'Lat	17.1 ± 3.7	17 ± 3.1	.89	18.1 ± 3	17.9 ± 2.8	.73	.93
E'Med	11.4 ± 2.2	12.2 ± 2.2	.11	12.5 ± 1.9	12.7 ± 2.1	.63	.24
E/e’ Med	7.6 ± 1.4	7.3 ± 1.4	.56	6.7 ± 1.3	6.6 ± 1.3	.7	.71
E/e’ Lat	5.1 ± 1.0	5.3 ± 1.1	.34	4.7 ± 1	4.7 ± 0.9	.75	.30

*Note*: Bolded values are those that reached statistical significance (*p* = .05).

Abbreviations: ARD, aortic root diameter; IVS, interventricular septal thickness; Lat, lateral; LAVI, left atrial volume index; LVEDD, left ventricular end diastolic diameter; LVEF, left ventricular ejection fraction; LVESD, left ventricular end systolic diameter; Med, medial; MV, mitral valve; PW, posterior wall thickness; RVID, right ventricular internal diameter; RWT, relative wall thickness; TAPSE, tricuspid annular plane systolic excursion.

^a^ Comparing the variance in echo parameters for Line vs. Non‐line positions.

^b^ Performed using Wilcoxon‐Ranks test as LVEF was non‐normally distributed.

## 4 DISCUSSION

In this study of collegiate American football players pre and postparticipation, we observed increased left and right ventricular chamber sizes, left atrial size, and aortic root diameter. There were no significant changes in parameters of wall thickness, diastolic function, or right ventricular systolic function (TAPSE). There was a decrease in LVEF in those with over 2 years of participation but the magnitude was small with questionable clinical significance. There was also no statistically significant change in any TTE parameter based on whether the athlete played a line position versus a non‐line position.

American football is classified as a IIB sport with a moderate dynamic component and moderate static component.^8^ With its emphasized strength component, effects on remodeling tend to favor wall thickness in some studies as opposed to chamber dilation found in more endurance athletics.^9^, ^10^ Our results deviated from this concept, showing chamber dilation more typical of endurance training but no significant change in wall thickness parameters. This has been shown before by Spence et al.^11^ using magnetic resonance imaging (MRI) data in which they followed a small cohort of endurance and resistance trained subjects longitudinally and showed increases in LV mass, end diastolic volume, and wall thickness in the endurance subjects but not in the resistance subjects. It is possible that the dynamic component of training was the dominant contributor to the morphologic changes observed in our population.

The most substantial change in this study was LAVI. LA size is a known risk factor for atrial fibrillation^12^ and some athletes are at higher risk for atrial arrhythmias, though this is largely felt to be at extremes of exercise.^13^, ^14^ Furthermore, the risk of atrial arrhythmias described is typically associated with endurance sports such as swimming, running, and cycling.^15^ McClean et al.^16^ also confirmed that in comparing extremes of athletes on the ACC classification, high dynamic low static athletes had significantly larger LA size compared to low dynamic high static athletes. American football, being a IIB classification with both moderate static and dynamic components falls in the middle of this spectrum and so again there is possibly a contribution of the dynamic component to left atrial size in this cohort of athletes.

In our study, there was a statistically significant decrease in LVEF from 60.7 ± 3.6 to 58.9 ± 2.8 (*p* = .007) observed only in the 34 athletes with more than 2 years of training. There were no athletes with post‐participation LVEF < 55% and thus no athletes were kept from participation or went on to have further testing such as cardiac MRI. Given the change is small, that both values are clinically normal and the change falls within a typical variability of sequential 2D echocardiography studies, this is likely clinically insignificant.^17^, ^18^ However, further prospective studies assessing response of LVEF to training programs of American football may be warranted.

This study had a number of limitations. As stated in the background and methods section, follow‐up echocardiograms were not performed for prospective assessment of cardiac remodeling specifically but instead were obtained as part of a mandatory return to participation protocol following COVID‐19. While we only included athletes with mild or asymptomatic COVID‐19, there may have been potential subclinical echocardiographic changes. Myocarditis related to COVID‐19 is rare, cited anywhere from less than 1%–4%, but nonetheless presents at least a small potential for bias.^19^, ^20^ During the peak of the pandemic, many patient encounters were performed utilizing telehealth strategies, and therefore we have incomplete follow‐up data on blood pressure in many of these athletes. Also, the data are retrospective in nature and as COVID‐19 echocardiograms were performed at different dates, there are heterogenous follow‐up times among athletes. Prospective trials will be needed in the future to better assess responses to training. As this study only included two collegiate football programs, sample size was relatively small. More widespread multicenter data would be desired in the future. Finally, training regimens likely differed by positions played and this was not controlled for in this study.

In conclusion, we found that participation in two collegiate American football programs was associated with changes in multiple echocardiographic parameters, including left atrial size, left and right ventricular diameters, and aortic root diameters. This cardiac remodeling more closely aligned with that typically seen in trained endurance athletes than strength athletes, which suggests a heterogeneity of training methods in American collegiate football.

## Supporting information

Supporting information.

## Data Availability

The data that support the findings of this study are available from the corresponding author upon reasonable request.
