# Supplementary material for: Longitudinal cardiac remodeling in collegiate American football players as assessed by echocardiography during their collegiate career
Source: Clin Cardiol. 2023 Aug 13;46(9):1090–6. doi: 10.1002/clc.24121 (PMC10540003; doi:10.1002/clc.24121)
Supplement: Supplementary file 1 — Supporting information. [file CLC-46-1090-s001.docx]

Supplementary Table. Echo parameters pre- and post-participation for at least one year (n=56).

|  | **Pre-participation** | **Post-participation** | **p-value** |
| --- | --- | --- | --- |
| **LVEF (%)** | 60.8 ± 3.7 | 59.6 ± 2.9 | **0.049*** |
| **LAVI (ml/m^2^)** | 26.5 ± 5.5 | 32.3 ± 9.0 | **<0.001** |
| **LVEDD (cm)** | 5.17 ± 0.45 | 5.32 ± 0.45 | **0.016** |
| **LVESD (cm)** | 3.39 ± 0.33 | 3.49 ± 0.35 | 0.053 |
| **LVEDI (cm)** | 2.26 ± 0.23 | 2.30 ± 0.23 | 0.068 |
| **PW (cm)** | 1.04 ± 0.13 | 1.07 ± 0.12 | 0.148 |
| **IVS (cm)** | 1.02 ± 0.13 | 1.02 ± 0.14 | 0.816 |
| **RWT** | 0.41 ± 0.06 | 0.40 ± 0.05 | 0.729 |
| **RVID (cm)** | 3.13 ± 0.63 | 3.84 ± 0.54 | **<0.001** |
| **TAPSE (mm)** | 24 ± 4 | 24 ± 3 | 0.978 |
| **ARD (cm)** | 3.06 ± 0.37 | 3.18 ± 0.36 | **0.015** |
| **Aortic root index (cm)** | 1.35 ± 0.15 | 1.39 ± 0.14 | **0.025** |
| **MV E (cm/s)** | 0.86 ± 0.16 | 0.85 ± 0.14 | 0.630 |
| **MV A (cm/s)** | 0.47 ± 0.13 | 0.43 ± 0.10 | **0.046** |
| **MV E/A** | 1.99 ± 0.69 | 2.10 ± 0.68 | 0.358 |
| **E’Lat (cm/s)** | 17.8 ± 3.1 | 17.5 ± 2.8 | 0.574 |
| **E’Med (cm/s)** | 12.2 ± 2.1 | 12.1 ± 1.9 | 0.728 |
| **E/e’ Med** | 7.0 ± 1.4 | 7.1 ± 1.3 | 0.543 |
| **E/e’ Lat** | 4.8 ± 1.0 | 5.0 ± 0.9 | 0.414 |

*Performed using Wilcoxon-Ranks Test as LVEF was non-normally distributed

LVEF, left ventricular ejection fraction; LAVI, left atrial volume index; LVEDD, left ventricular end diastolic diameter; LVESD, left ventricular end systolic diameter; LVEDI, LV end diastolic index, PW, posterior wall thickness; IVS, interventricular septal thickness; RWT, relative wall thickness; RVID, right ventricular internal diameter; TAPSE, tricuspid annular plane systolic excursion; ARD, aortic root diameter; MV, mitral valve; Lat, lateral; Med, medial.
